# Supplementary material for: Acceptance and Adherence to COVID-19 Vaccination—The Role of Cognitive and Emotional Representations
Source: Int J Environ Res Public Health. 2022 Jul 28;19(15):9268. doi: 10.3390/ijerph19159268 (PMC9368462; doi:10.3390/ijerph19159268)
Supplement: Supplementary file 1 [file ijerph-19-09268-s001.zip › Supplementary File I - CHERRIES checklist.pdf]

Supplementary File SI. Checklist for Reporting Results of Internet E-Surveys (CHERRIES) .

Item Category	Explanation		Page
**Design**	The study involved a convenience sample. Eligibility criteria included being 18 years of age or older, residing in Portugal and being able to complete the survey in Portuguese.		
IRB	**Approval**:	The study has been approved by the Hospital of S. João, Porto review boards and ethics committee	
	**Informed consent**:	Before completing the questionnaire, all participants provided electronic informed consent.	
	**Data protection**:	No information that allows for personal identification was collected.	
Development and pre-testing	The survey was developed by a multidisciplinary team and based on the Self-Regulation Model [18] and the Necessity-Concerns Framework [19].		
Recruitment process	**Survey type**:	The data was collected using an open survey.	
	**Contact mode**:	Initial contact with participants was made on the Internet, through email or social media.	
	**Advertising the survey:**	Social media (official faculty pages) and several online newspapers	
Survey administration	**Web/E-mail**:	The survey was input on an online survey platform – LimeSurvey. Data was entered automatically when participants responded to the questions.	
	**Context**:	The survey was disseminated using social media (official faculty pages) and several online newspapers	
	**Mandatory/voluntary:**	The survey was voluntary.	
	**Incentives**:	None.	
	**Time/Date:**	27th December 2020 to 27th January 2021	
	**Randomization of items or questionnaire**:	N/A	
	**Adaptive questioning**:	Some questions only were displayed if certain answers were previously selected.	
	**Number of items**:	16	
	**Number of screens**:	11	
	**Completeness check**:	N/A	
	**Review step:**	Yes	
Response rates	**Unique site visitor**:	N/A	
	**View rate**:	N/A	
	**Participation rate**:	N/A	
	**Completion rate**:	N/A	
Preventing multiple entries	**Cookies used**:	Not used	
	**IP check**:	Not used	
	**Log file analysis**:	Not used	
	**Registration**:	N/A	
Analysis	**Handling of incomplete surveys**:	Only completed surveys were analysed.	
	**Questionnaires submitted with an atypical timestamp**:	N/A	
	**Statistical correction:**	N/A	
