# Supplementary material for: Acceptance and Adherence to COVID-19 Vaccination—The Role of Cognitive and Emotional Representations
Source: Int J Environ Res Public Health. 2022 Jul 28;19(15):9268. doi: 10.3390/ijerph19159268 (PMC9368462; doi:10.3390/ijerph19159268)
Supplement: Supplementary file 1 [file ijerph-19-09268-s001.zip › Supplementary File II - Questionnaire.pdf]

A1. Ao assinalar 'SIM' estará a aceitar participar voluntariamente no estudo, permitindo a utilização dos dados fornecidos com o propósito de aumentar o conhecimento científico, confiando que estes apenas serão utilizados para esta investigação e nas garantias de confidencialidade e anonimato.

- SIM, aceito participar no estudo de acordo com a informação acima facultada. ☐
- NÃO pretendo participar no estudo. ☐

B1. Sexo:

- Masculino ☐
- Feminino ☐
- Outro ☐

B2. Idade:

☐☐☐☐☐☐☐☐☐☐

B3. Escolaridade:

- Ensino primário (até ao 4º ano) ☐
- Ensino básico (até ao 9º ano) ☐
- Ensino secundário (até ao 12º ano) ☐
- Licenciatura ☐
- Mestrado ☐
- Doutoramento ☐

B4. Situação Laboral:

- Estudante ☐
- Trabalhador(a) dependente ☐
- Trabalhador(a) independente ☐
- Doméstica(o) ☐
- Desempregado(a) ☐
- Reformado(a) ☐

B5. Número de pessoas com quem vive:

☐☐☐☐☐☐☐☐☐☐

B6. Distrito de Residência:

- Aveiro ☐
- Beja ☐
- Braga ☐
- Bragança ☐
- Castelo Branco ☐
- Coimbra ☐
- Évora ☐
- Faro ☐
- Guarda ☐
- Leiria ☐
- Lisboa ☐
- Portalegre ☐
- Porto ☐
- Santarém ☐
- Setúbal ☐
- Viana do Castelo ☐
- Vila Real ☐
- Viseu ☐
- Região Autónoma da Madeira ☐
- Região Autónoma dos Açores ☐

C1. Está infetado(a) com a COVID-19?

- Sim ☐
- Não ☐

C2. **Já esteve infetado(a) com a COVID-19?**

Sim ☐
Não ☐

D1. **Por favor responda às seguintes questões de acordo com a forma como está a viver a sua atual situação de doença:**

	Nada	Pouco	Moderadamente	Muito	Extremamente	Não sei
Quanto é que a COVID-19 afeta a sua vida?	☐	☐	☐	☐	☐	☐
Quão capaz se sente de controlar a COVID-19?	☐	☐	☐	☐	☐	☐
Quanto o(a) afetam os sintomas da COVID-19?	☐	☐	☐	☐	☐	☐
Quanto o(a) afeta emocionalmente a COVID-19?	☐	☐	☐	☐	☐	☐

E1. **Por favor responda as seguintes questões de acordo com a forma como viveu a situação de doença que teve:**

	Nada	Pouco	Moderadamente	Muito	Extremamente	Não sei
Quanto é que a COVID-19 afetou a sua vida?	☐	☐	☐	☐	☐	☐
Quão capaz se sentiu de controlar a COVID-19?	☐	☐	☐	☐	☐	☐
Quanto o(a) afetaram os sintomas da COVID-19?	☐	☐	☐	☐	☐	☐
Quanto o(a) afetou emocionalmente a COVID-19?	☐	☐	☐	☐	☐	☐

F1. **Por favor responda às seguintes questões em relação à pandemia da COVID-19:**

	Nada	Pouco	Moderadamente	Muito	Extremamente	Não sei
Quanto é que a pandemia da COVID-19 tem afetado a	☐	☐	☐	☐	☐	☐
Quão preocupado(a) está em ser infetado(a) com a	☐	☐	☐	☐	☐	☐
Quão informado(a) está sobre a pandemia da	☐	☐	☐	☐	☐	☐
Quanto é que a situação pandémica da COVID-19 o(a)	☐	☐	☐	☐	☐	☐

G1. **Já foi vacinado(a) para a COVID-19?**

Sim ☐
Não ☐

H1. **Aceita a vacina para a COVID-19, se disponível?**

Sim ☐
Não ☐

H2. **Aceita a vacina da COVID-19, se recomendada no seu emprego?**

Sim ☐
Não ☐

I1. **Por favor responda às seguintes questões:**

	Nada	Pouco	Moderadamente	Muito	Extremamente	Não sei
Quão eficaz pensa ser uma vacina para a prevenção da	☐	☐	☐	☐	☐	☐
Quão preocupado(a) está com a toma de uma vacina para	☐	☐	☐	☐	☐	☐
Quão informado(a) está sobre vacinas da COVID-19?	☐	☐	☐	☐	☐	☐
Quanto é que a toma da vacina para a COVID-19 o(a)	☐	☐	☐	☐	☐	☐

J1. **Por favor responda às seguintes questões:**

	Discordo Totalmente	Discordo	Não Concordo nem Discordo	Concordo	Concordo Totalmente	Não sei
A minha saúde no futuro dependerá da vacina da	☐	☐	☐	☐	☐	☐
A minha vida será impossível sem a vacina da	☐	☐	☐	☐	☐	☐
Sem a vacina da COVID-19 eu ficarei muito doente.	☐	☐	☐	☐	☐	☐
Ter que tomar a vacina da COVID-19 preocupa-me.	☐	☐	☐	☐	☐	☐
Preocupo-me acerca dos efeitos da vacina da COVID-19.	☐	☐	☐	☐	☐	☐
A vacina da COVID-19 é um mistério para mim.	☐	☐	☐	☐	☐	☐

Obrigado pela sua colaboração. Se necessitar de algum esclarecimento adicional, pode entrar em contacto com os investigadores responsáveis, através do e-mail: grupoatfmup@gmail.com.

A1. By checking the YES box, you are accepting to participate voluntarily in our study, allowing us to use the data you provide with the purpose of furthering scientific knowledge, and trusting that all data will be confidential, anonymized and will only be used for this study's purposes.

☐ YES, I want to participate in the study and fully acknowledge all the information provided above.

☐ NO, I do not want to participate in this study.

B1. Gender:

☐ Male

☐ Female

☐ Other

B2. Age: ☐

B3. Education Level:

☐ Primary Education

☐ Basic Education

☐ Secondary Education

☐ Bachelorate degree

☐ Master's degree

☐ PhD

B4. Current Employment Status:

- ☐ Student
- ☐ Dependent worker
- ☐ Independent worker
- ☐ Housewife/husband
- ☐ Unemployed

B5. Number of Cohabitants: ☐

B6. District Where You Reside:

- ☐ Aveiro
- ☐ Beja
- ☐ Braga
- ☐ Bragança
- ☐ Castelo Branco
- ☐ Coimbra
- ☐ Évora
- ☐ Faro
- ☐ Guarda
- ☐ Leiria
- ☐ Lisboa
- ☐ Portalegre
- ☐ Porto
- ☐ Santarém
- ☐ Setúbal
- ☐ Viana do Castelo
- ☐ Vila Real
- ☐ Viseu
- ☐ Região Autónoma da Madeira
- ☐ Região Autónoma dos Açores

C1. Are you currently infected with COVID-19?

☐ Yes.

☐ No.

C2. Have you ever been infected with COVID-19?

☐ Yes.

☐ No.

D1. Please answer the following questions according to the way you're living your current situation as a patient:

	Nothing	Little	Moderately	A lot	Extremely	I do not know
How much is COVID-19 affecting your life?						
How capable of controlling COVID-19 do you feel?						
How much do COVID-19 symptoms affect you?						
How much does COVID-19 affect you emotionally?						

E1. Please answer the following questions according to the way you lived your situation as a patient:

	Nothing	Little	Moderately	A lot	Extremely	I do not know
How much did COVID-19 affect your life?						
How capable of controlling COVID-19 did you feel?						
How much did COVID-19 symptoms affect you?						
How much did COVID-19 affect you emotionally?						

F1. Please answer the following questions about the COVID-19 pandemic:

	Nothing	Little	Moderately	A lot	Extremely	I do not know
How much has the COVID-19 pandemic affected your life?						
How worried about being infected with COVID-19 are you?						
How informed about the COVID-19 pandemic are you?						
How much did the COVID-19 pandemic affect you emotionally?						

G1. Have you taken a COVID-19 vaccine?

☐ Yes.

☐ No.

H1. If available, would you accept a COVID-19 vaccine?

☐ Yes.

☐ No.

H2. If recommended at your workplace, would you accept a COVID-19 vaccine?

☐ Yes.

☐ No.

I1. Please answer the following questions about COVID-19 vaccination:

	Nothing	Little	Moderately	A lot	Extremely	I do not know
How effective in do you think COVID-19 vaccination is?						
How worried about taking a COVID-19 vaccine are you?						
How informed about the COVID-19 vaccines are you?						
How much does taking a COVID-19 vaccine affect you emotionally?						

J1. Please choose the best option regarding your agreement with the following sentences:

	Completely Disagree	Disagree	Neither Agree nor Disagree	Agree	Completely Agree	I do not know
My future health will depend on COVID-19 vaccination.						
My life will be impossible without a COVID-19 vaccine.						
Without a COVID-19 vaccine, I will be very ill.						
Taking a COVID-19 vaccine makes me worried.						
I am worried about the effects of COVID-19 vaccination.						
COVID-19 vaccines are a mystery to me.						
