# Supplementary material for: Acceptance and Adherence to COVID-19 Vaccination—The Role of Cognitive and Emotional Representations
Source: Int J Environ Res Public Health. 2022 Jul 28;19(15):9268. doi: 10.3390/ijerph19159268 (PMC9368462; doi:10.3390/ijerph19159268)
Supplement: Supplementary file 1 [file ijerph-19-09268-s001.zip › Supplementary Tables.pdf]

Supplementary Tables S1. Comparison of COVID-19 and COVID-19 vaccination perspectives between sexes.

	Sex				
	Female		Male		p-value
**How much has the COVID-19 pandemic affected your life?**					**<0.001**
Extremely	1.052	(47.8%)	448	(50.9%)	
Moderately	1.090	(49.5%)	385	(43.7%)	
Little/Not at all	60	(2.7%)	48	(5.4%)	
**How worried are you about becoming infected with COVID-19?**					**<0.001**
Extremely	1.165	(52.9%)	457	(51.8%)	
Moderately	905	(41.1%)	308	(34.9%)	
Little/Not at all	132	(6.0%)	117	(13.3%)	
**How informed are you about the COVID-19 pandemic?**					0.692
Extremely	908	(41.5%)	369	(42.0%)	
Moderately	1.275	(58.2%)	505	(57.5%)	
Little/Not at all	6	(0.3%)	4	(0.5%)	
**How much does the COVID-19 pandemic affect you emotionally?**					**<0.001**
Extremely	1.191	(54.1%)	440	(49.9%)	
Moderately	802	(36.4%)	268	(30.4%)	
Little/Not at all	209	(9.5%)	174	(19.7%)	
**How efficacious do you think vaccination is in preventing COVID-19 infection?**					0.975
Extremely	868	(43.8%)	354	(43.3%)	
Moderately	1.038	(52.3%)	430	(52.6%)	
Little/Not at all	78	(3.9%)	33	(4.0%)	
**How worried are you about taking a COVID-19 vaccine?**					**<0.001**
Extremely	725	(35.2%)	229	(27.0%)	
Moderately	271	(13.2%)	95	(11.2%)	
Little/Not at all	1.063	(51.6%)	523	(61.7%)	
**How informed are you about COVID-19 vaccines?**					0.802
Extremely	1.156	(56.3%)	469	(55.4%)	
Moderately	723	(35.2%)	308	(36.4%)	
Little/Not at all	176	(8.6%)	69	(8.2%)	
**How much would taking a COVID-19 vaccine affect you emotionally?**					**0.045**
Extremely	622	(31.4%)	219	(27.0%)	
Moderately	330	(16.6%)	132	(16.3%)	

Little/Not at all	1.031	(52.0%)	461	(56.8%)	
**My future health will depend on COVID-19 vaccination.**					**0.016**
Completely agree/Agree	1.040	(54.4%)	459	(56.8%)	
Neither agree nor disagree	522	(27.3%)	180	(22.3%)	
Disagree/Completely disagree	349	(18.3%)	169	(20.9%)	
**My life will be impossible without a COVID-19 vaccine.**					0.259
Completely agree/Agree	446	(23.0%)	198	(24.4%)	
Neither agree nor disagree	503	(25.9%)	187	(23.0%)	
Disagree/Completely disagree	990	(51.1%)	428	(52.6%)	
**Without a COVID-19 vaccine, I will become very sick.**					0.574
Completely agree/Agree	198	(11.1%)	76	(9.9%)	
Neither agree nor disagree	595	(33.3%)	252	(32.7%)	
Disagree/Completely disagree	993	(55.6%)	442	(57.4%)	
**Taking a COVID-19 vaccine worries me.**					**<0.001**
Completely agree/Agree	452	(22.2%)	124	(14.8%)	
Neither agree nor disagree	238	(11.7%)	102	(12.1%)	
Disagree/Completely disagree	1.342	(66.0%)	614	(73.1%)	
**I'm worried about COVID-19 vaccination's effects.**					**<0.001**
Completely agree/Agree	917	(45.7%)	299	(35.6%)	
Neither agree nor disagree	354	(17.6%)	147	(17.5%)	
Disagree/Completely disagree	736	(36.7%)	393	(46.8%)	
**COVID-19 vaccines are a mystery to me.**					**0.041**
Completely agree/Agree	293	(14.4%)	92	(11.0%)	
Neither agree nor disagree	346	(17.0%)	155	(18.5%)	
Disagree/Completely disagree	1.391	(68.5%)	593	(70.6%)	

Chi-squared test.

Supplementary Tables S2. Comparison of COVID-19 and COVID-19 vaccination perspectives in different education level categories.

	Superior		Secondary School		Primary School		p-value
**How worried are you about becoming infected with COVID-19?**							**0.001**
Extremely	1.265	(53.5%)	326	(52.0%)	33	(34.0%)	
Moderately	887	(37.5%)	270	(43.1%)	57	(58.8%)	
Little/Not at all	211	(8.9%)	31	(4.9%)	7	(7.2%)	
**How much does the COVID-19 pandemic affect you emotionally?**							**0.039**
Extremely	1.260	(53.3%)	335	(53.3%)	37	(38.5%)	
Moderately	804	(34.0%)	220	(35.0%)	47	(49.0%)	
Little/Not at all	298	(12.6%)	74	(11.8%)	12	(12.5%)	
**How informed are you about COVID-19 vaccines?**							**<0.001**
Extremely	1.229	(55.9%)	355	(57.9%)	41	(44.6%)	

Moderately	820	(37.3%)	190	(31.0%)	23	(25.0%)	
Little/Not at all	150	(6.8%)	68	(11.1%)	28	(30.4%)	
**My life will be impossible without a COVID-19 vaccine.**							**0.048**
Completely agree/Agree	483	(22.8%)	139	(24.7%)	22	(28.6%)	
Neither agree nor disagree	514	(24.3%)	150	(26.7%)	26	(33.8%)	
Disagree/Completely disagree	1.119	(52.9%)	273	(48.6%)	29	(37.7%)	
**Without a COVID-19 vaccine, I will become very sick.**							**0.008**
Completely agree/Agree	193	(9.9%)	66	(12.4%)	15	(20.5%)	
Neither agree nor disagree	638	(32.6%)	182	(34.3%)	27	(37.0%)	
Disagree/Completely disagree	1.124	(57.5%)	283	(53.3%)	31	(42.5%)	
**Taking a COVID-19 vaccine worries me.**							**0.002**
Completely agree/Agree	436	(20.0%)	118	(19.6%)	23	(25.8%)	
Neither agree nor disagree	237	(10.8%)	85	(14.1%)	19	(21.3%)	
Disagree/Completely disagree	1.512	(69.2%)	398	(66.2%)	47	(52.8%)	
**I'm worried about COVID-19 vaccination's effects.**							**<0.001**
Completely agree/Agree	906	(41.8%)	259	(43.3%)	53	(62.4%)	
Neither agree nor disagree	368	(17.0%)	114	(19.1%)	19	(22.4%)	
Disagree/Completely disagree	892	(41.2%)	225	(37.6%)	13	(15.3%)	
**COVID-19 vaccines are a mystery to me.**							**<0.001**
Completely agree/Agree	250	(11.5%)	103	(17.1%)	34	(38.2%)	
Neither agree nor disagree	349	(16.0%)	133	(22.1%)	19	(21.3%)	
Disagree/Completely disagree	1.583	(72.5%)	366	(60.8%)	36	(40.4%)	

Chi-squared test.

Supplementary Tables S3. Comparison of COVID-19 and COVID-19 vaccination perspectives in different age categories.

	**18-24**		**25-34**		**35-44**		**45-54**		**55-64**		**>64**		**p-value**
**How much has the COVID-19 pandemic affected your life?**													0.059
Extremely	278	(48.6%)	251	(49.2%)	360	(49.3%)	272	(48.2%)	194	(44.6%)	144	(53.3%)	
Moderately	283	(49.5%)	235	(46.1%)	351	(48.1%)	269	(47.7%)	224	(51.5%)	112	(41.5%)	
Little/Not at all	11	(1.9%)	24	(4.7%)	19	(2.6%)	23	(4.1%)	17	(3.9%)	14	(5.2%)	
**How worried are you about becoming infected with COVID-19?**													**<0.001**
Extremely	317	(55.5%)	283	(55.4%)	408	(55.8%)	291	(51.5%)	197	(45.3%)	126	(46.8%)	
Moderately	199	(34.9%)	177	(34.6%)	263	(36.0%)	234	(41.4%)	216	(49.7%)	124	(46.1%)	
Little/Not at all	55	(9.6%)	51	(10.0%)	60	(8.2%)	40	(7.1%)	22	(5.1%)	19	(7.1%)	
**How informed are you about the COVID-19 pandemic?**													-
Extremely	253	(44.8%)	233	(45.8%)	303	(41.5%)	226	(40.2%)	168	(38.8%)	92	(34.6%)	
Moderately	310	(54.9%)	272	(53.4%)	426	(58.4%)	335	(59.6%)	264	(61.0%)	172	(64.7%)	
Little/Not at all	2	(0.4%)	4	(0.8%)	1	(0.1%)	1	(0.2%)	1	(0.2%)	2	(0.8%)	
**How much does the COVID-19 pandemic affect you emotionally?**													0.587
Extremely	299	(52.3%)	269	(52.6%)	386	(52.8%)	304	(53.8%)	227	(52.3%)	145	(53.9%)	
Moderately	210	(36.7%)	165	(32.3%)	265	(36.3%)	192	(34.0%)	150	(34.6%)	87	(32.3%)	

Little/Not at all	63	(11.0%)	77	(15.1%)	80	(10.9%)	69	(12.2%)	57	(13.1%)	37	(13.8%)	
**How efficacious do you think vaccination is in preventing COVID-19 infection?**													**<0.001**
Extremely	268	(48.7%)	211	(46.1%)	305	(46.8%)	209	(42.7%)	154	(38.9%)	75	(29.5%)	
Moderately	277	(50.4%)	218	(47.6%)	319	(48.9%)	250	(51.1%)	229	(57.8%)	172	(67.7%)	
Little/Not at all	5	(0.9%)	29	(6.3%)	28	(4.3%)	30	(6.1%)	13	(3.3%)	7	(2.8%)	
**How worried are you about taking a COVID-19 vaccine?**													**<0.001**
Extremely	123	(21.9%)	118	(25.2%)	238	(34.8%)	202	(38.8%)	173	(42.5%)	99	(37.6%)	
Moderately	52	(9.3%)	50	(10.7%)	107	(15.7%)	73	(14.0%)	53	(13.0%)	32	(12.2%)	
Little/Not at all	387	(68.9%)	301	(64.2%)	338	(49.5%)	246	(47.2%)	181	(44.5%)	132	(50.2%)	
**How informed are you about COVID-19 vaccines?**													0.616
Extremely	302	(53.9%)	266	(57.1%)	392	(57.2%)	290	(55.8%)	234	(57.4%)	139	(53.5%)	
Moderately	211	(37.7%)	162	(34.8%)	226	(33.0%)	182	(35.0%)	147	(36.0%)	102	(39.2%)	
Little/Not at all	47	(8.4%)	38	(8.2%)	67	(9.8%)	48	(9.2%)	27	(6.6%)	19	(7.3%)	
**How much would taking a COVID-19 vaccine affect you emotionally?**													**0.004**
Extremely	150	(28.6%)	132	(29.1%)	197	(29.8%)	172	(34.1%)	123	(31.4%)	66	(25.7%)	
Moderately	99	(18.9%)	64	(14.1%)	131	(19.8%)	85	(16.8%)	50	(12.8%)	33	(12.8%)	
Little/Not at all	276	(52.6%)	258	(56.8%)	332	(50.3%)	248	(49.1%)	219	(55.9%)	158	(61.5%)	
**My future health will depend on COVID-19 vaccination.**													**0.001**
Completely agree/Agree	330	(60.3%)	232	(51.3%)	321	(49.7%)	256	(53.6%)	204	(56.7%)	154	(65.5%)	
Neither agree nor disagree	128	(23.4%)	126	(27.9%)	186	(28.8%)	125	(26.2%)	94	(26.1%)	43	(18.3%)	
Disagree/Completely disagree	89	(16.3%)	94	(20.8%)	139	(21.5%)	97	(20.3%)	62	(17.2%)	38	(16.2%)	
**My life will be impossible without a COVID-19 vaccine.**													**<0.001**
Completely agree/Agree	119	(21.4%)	84	(18.4%)	119	(18.3%)	125	(25.8%)	121	(32.6%)	76	(32.3%)	
Neither agree nor disagree	134	(24.1%)	117	(25.7%)	178	(27.4%)	116	(24.0%)	83	(22.4%)	61	(26.0%)	
Disagree/Completely disagree	302	(54.4%)	255	(55.9%)	353	(54.3%)	243	(50.2%)	167	(45.0%)	98	(41.7%)	
**Without a COVID-19 vaccine, I will become very sick.**													**<0.001**
Completely agree/Agree	25	(4.7%)	24	(5.6%)	50	(8.3%)	62	(13.9%)	66	(19.2%)	47	(23.5%)	
Neither agree nor disagree	154	(28.7%)	135	(31.7%)	228	(37.9%)	147	(33.0%)	119	(34.6%)	63	(31.5%)	
Disagree/Completely disagree	358	(66.7%)	267	(62.7%)	324	(53.8%)	237	(53.1%)	159	(46.2%)	90	(45.0%)	
**Taking a COVID-19 vaccine worries me.**													**<0.001**
Completely agree/Agree	56	(9.9%)	73	(15.8%)	171	(25.3%)	138	(27.0%)	81	(20.3%)	58	(22.5%)	
Neither agree nor disagree	62	(11.0%)	44	(9.5%)	94	(13.9%)	66	(12.9%)	47	(11.8%)	27	(10.5%)	
Disagree/Completely disagree	445	(79.0%)	346	(74.7%)	412	(60.9%)	307	(60.1%)	271	(67.9%)	173	(67.1%)	
**I'm worried about COVID-19 vaccination's effects.**													**<0.001**
Completely agree/Agree	169	(29.9%)	170	(36.9%)	341	(50.3%)	271	(53.7%)	162	(42.1%)	104	(41.4%)	
Neither agree nor disagree	87	(15.4%)	76	(16.5%)	122	(18.0%)	84	(16.6%)	80	(20.8%)	51	(20.3%)	
Disagree/Completely disagree	309	(54.7%)	215	(46.6%)	215	(31.7%)	150	(29.7%)	143	(37.1%)	96	(38.2%)	
**COVID-19 vaccines are a mystery to me.**													**<0.001**
Completely agree/Agree	34	(6.0%)	48	(10.4%)	104	(15.4%)	94	(18.3%)	72	(18.1%)	35	(13.7%)	
Neither agree nor disagree	60	(10.6%)	79	(17.1%)	136	(20.1%)	108	(21.0%)	71	(17.8%)	47	(18.4%)	
Disagree/Completely disagree	470	(83.3%)	334	(72.5%)	437	(64.5%)	312	(60.7%)	255	(64.1%)	174	(68.0%)	

Chi-squared test.
